# 1,3-Substituted Imidazolidine-2,4,5-triones: Synthesis and Inhibition of Cholinergic Enzymes

**DOI:** 10.3390/molecules16097565

**Published:** 2011-09-05

**Authors:** Vladimir Pejchal, Sarka Stepankova, Zdenka Padelkova, Ales Imramovsky, Josef Jampilek

**Affiliations:** 1 Institute of Organic Chemistry and Technology, Faculty of Chemical Technology, University of Pardubice, Studentska 573, 532 10 Pardubice, Czech Republic; 2 Department of Biological and Biochemical Sciences, Faculty of Chemical Technology, University of Pardubice, Studentska 573, Pardubice 53210, Czech Republic; 3 Department of General and Inorganic Chemistry, Faculty of Chemical Technology, University of Pardubice, Studentska 573, 532 10 Pardubice, Czech Republic; 4 Department of Chemical Drugs, Faculty of Pharmacy, University of Veterinary and Pharmaceutical Sciences, Palackeho 1/3, 612 42 Brno, Czech Republic

**Keywords:** 1-(aryl)-3-[(*R*)-1-(6-fluorobenzo[*d*]thiazol-2-yl)ethyl]imidazolidine-2,4,5-triones, X-ray diffraction, lipophilicity, *in vitro* acetylcholinesterase inhibition, *in vitro* butyrylcholinesterase inhibition, structure-activity relationships

## Abstract

A series of novel and highly active acetylcholinesterase and butyrylcholinesterase inhibitors derived from substituted benzothiazoles containing an imidazolidine-2,4,5-trione moiety were synthesized and characterized. The molecular structure of 1-(2,6-diisopropyl-phenyl)-3-[(1*R*)-1-(6-fluoro-1,3-benzothiazol-2-yl)ethyl]-imidazolidine-2,4,5-trione (**3g**) was determined by single-crystal X-ray diffraction. Both optical isomers are present as two independent molecules in the triclinic crystal system. The lipophilicity of the compounds was determined as the partition coefficient log K_ow_ using the traditional shake-flask method. The *in vitro* inhibitory activity on acetylcholinesterase from electric eel and butyrylcholinesterase isolated from equine serum was determined. The inhibitory activity on acetylcholinesterase was significantly higher than that of the standard drug rivastigmine. The discussed compounds are also promising inhibitors of butyrylcholinesterase, as some of the prepared compounds inhibit butyrylcholinesterase better than the internal standards rivastigmine and galanthamine. The highest inhibitory activity (IC_50_ = 1.66 μmol/L) corresponds to the compound 1-(4-isopropylphenyl)-3-[(*R*)-1-(6-fluorobenzo[*d*]thiazol-2-yl)ethyl]imidazolidine-2,4,5-trione (**3d**). For all the studied compounds, the relationships between the lipophilicity and the chemical structure as well as their structure-activity relationships are discussed.

## 1. Introduction

Substituted benzothiazoles are heterocyclic systems with a wide range of interesting biological activities. The substituents of these compounds on the 2-position have been found to be the most important modulators of the bio-activity of this type of compounds. Diverse biological activities such as antibacterial [1], fungicidal [2] and anticancer [3,4] were described. Benzothiazoles and benzoxazoles are also described as potent inhibitors of various enzymes such as 5-lipoxygenase [5], cyclooxygenase [6], aldose and aldehyde reductase [7], serine hydrolases [8,9] or thrombin inhibitors, known as coagulation factor II (F2) [10]. Generally it can be noted that benzothiazoles can serve as unique and versatile scaffolds for experimental drug design.

The group of serine hydrolases includes two important enzymes: Acetylcholinesterase (AChE, EC 3.1.1.7) and butyrylcholinesterase (BChE, EC 3.1.1.8) also known as pseudocholinesterase. These serine hydrolases belong structurally to the class of proteins known as the esterase/lipase family, within the α/β-hydrolase fold superfamily [11]. The major role of AChE is to catalyze the hydrolysis of acetylcholine (ACh) in cholinergic synapses, whereas the function of BChE is less clearly defined because it can hydrolyze ACh as well as other esters [12]. AChE and BChE inhibitors are used in treatment of various neuromuscular disorders and have provided the first generation of drugs for treatment of Alzheimer’s disease (AD) [13], which is a progressive physical disorder which causes increasingly severe impairment in the cognitive and functional ability of individuals suffering from the disease [14]. It is a degenerative disease of the brain that leads to the conditions collectively called dementia [15]. The inhibition of AChE and BChE is directly connected with treatment of AD. The cholinergic hypothesis proposes that AD is caused by reduced synthesis of the neurotransmitter acetylcholine [16]. The inhibition of the mentioned enzymes causes an increase in the concentration of acetylcholine in cholinergic synapses, which results in alleviation of the disease. New and potent AChE inhibitors may be helpful in the treatment of this disease.

The basic 2-substituted-1,3-benzothiazole scaffold is essential for some antimicrobial compounds [17], herbicides, plant desiccants and defoliant compounds [18]. Isopropyl [(*S*)-1-[(*R*)-1-(6-flourobenzothiazolel-2-yl)ethylcarbamoyl]-2-methylpropyl] carbamate is a commercially used fungicide which is effective for controlling the oomycete fungal pathogen *Plasmopara viticola*, which causes downy mildew in grapevines [19]. Position 2 of benzothiazoles is the most suitable for affecting physico-chemical properties of these compounds. The aim of this study was the modification of the amino moiety leading to the synthesis of substituted (6-fluorobenzo[*d*]thiazol-2-yl)ethanamines with incorporated 2,4,6-trioxoimidazolidine moieties as potent inhibitors of AChE and BChE. The five-member 2,4,6-trioxoimidazolidine ring (a stabilized urea) can be understood as an isostere of a carbamate moiety [20].

Many low-molecular-weight drugs cross biological membranes through passive transport, which strongly depends on their lipophilicity, therefore the experimental log *P* (or log K_ow_) *n*-octanol/water partition coefficients were determined. Structure-activity relationships between the chemical structure, physical properties and biological activities of the evaluated compounds are discussed.

## 2. Results and Discussion

### 2.1. Chemistry

**Scheme 1:**
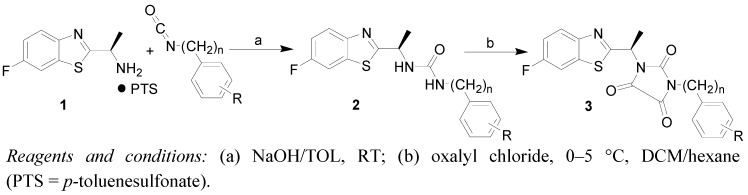
Synthesis of 1,3-substituted imidazolidine-2,4,5-triones **3a**–**k**.

The synthesis of 1-(aryl)-3-[(*R*)-1-(6-fluorobenzo[*d*]thiazol-2-yl)ethyl]imidazolidine-2,4,5-triones **3a**–**k** was accomplished according to Scheme 1. The starting compound (*R*)-1-(6-fluorobenzo[*d*]thiazol-2-yl)ethanamine (**1**) was prepared as the corresponding *p*-toluenesulfonate salt (PTS) according to a literature method [21]. Synthesis of the key intermediates disubstituted ureas **2a**–**k** was recently described [9].

The desired compounds **3a**–**k** were synthesized in dichloromethane (DCM), where the starting disubstituted ureas were treated with oxalyl chloride at low temperature (0–5 °C). The final products were precipitated from the reaction mixture by addition of *n*-hexane. The purification of the prepared compounds was realized by dissolving in DCM, and adding *n*-hexane to induce precipitation of the products in high yields (80%–90%). Products **3a**–**k** were characterized by their melting points, ^1^H-, ^13^C-, ^19^F-NMR, IR spectroscopy and elemental analyses (CHN).

### 2.2. Crystallography

**Figure 1:**
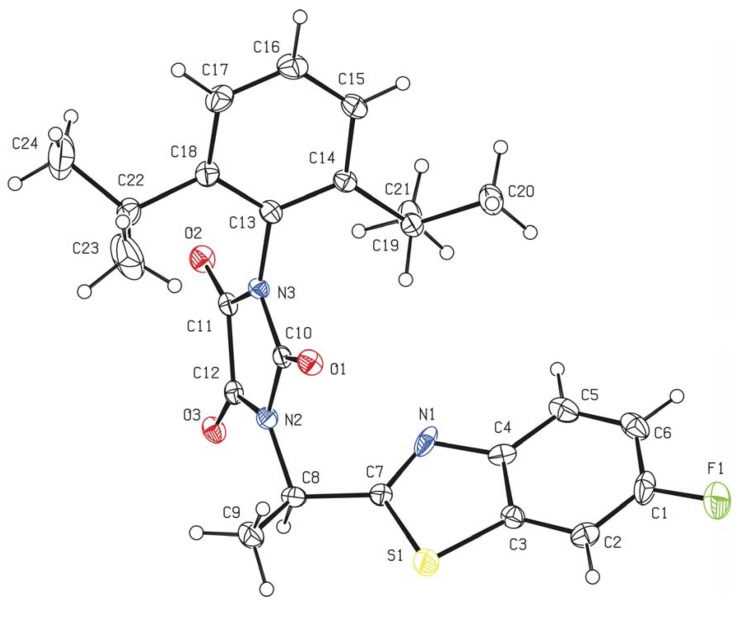
Molecular structure (ORTEP 30% probability level) of compound **3g**, only one of two independent molecules is shown. Selected interatomic distances [Å] and bond angles [°]: O1 C10 1.199(5), O2 C11 1.203(5), O3 C12 1.197(5), N2 C10 1.407(5), C10 N3 1.404(5), N3 C11 1.376(5), C11 C12 1.542(6), C12 N2 1.373(5), S1 C3 1.727(4), C3 C4 1.400(6), C4 N1 1.409(6), N1 C7 1.350(6), C7 S1 1.751(4); O1 C10 N3 126.8(4), O1 C10 N2 126.0(4), O2 C11 N3 127.6(4), O2 C11 C12 126.5(4), O3 C12 C11 127.4(4), O3 C12 N2 128.1(4), N2 C10 N3 107.2(3), C10 N3 C11 110.7(3), N3 C11 C12 105.9(3), C11 C12 N2 104.5(3), C12 N2 C10 111.7(3), C3 S1 C7 88.1(2), S1 C3 C4 111.0(3), C4 N1 C7 106.9(4), N1 C7 S1 117.8(3), N2 C8 C7 108.2(3).

The exemplary compound **3g** crystallizes in the triclinic crystal system, achiral point group *P*-1 with two independent molecules (different enantiomers) within the unit cell (Figure 1). The opposite orientation of the C8 atom seems to be the only remarkable difference between these two molecules. The interatomic distances and angles including torsion and interplanar angles are similar to the typical values found in the literature for similar atom combinations [22].

Although cyclic ureas such as, for example, hydantoins or alantoins, are frequently studied, there are only ten examples of 3,4-dicarbonyl bridged ureas [23,24,25,26,27,28,29,30,31,32,33,34], and there are only four structures that are similar to the structure of compound **3g** where the diazacyclopentane ring is 3,4-dicarbonyl substituted and nitrogen atoms are not of the NH type [35,36]. The determined structure of compound **3g** is the first asymmetric cyclic urea within the set.

**Figure 2:**
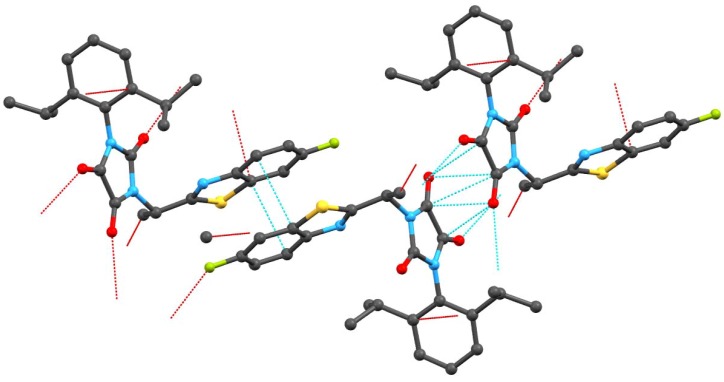
π-π Stacking interactions of aromatic rings and C=O groups.

**Figure 3:**
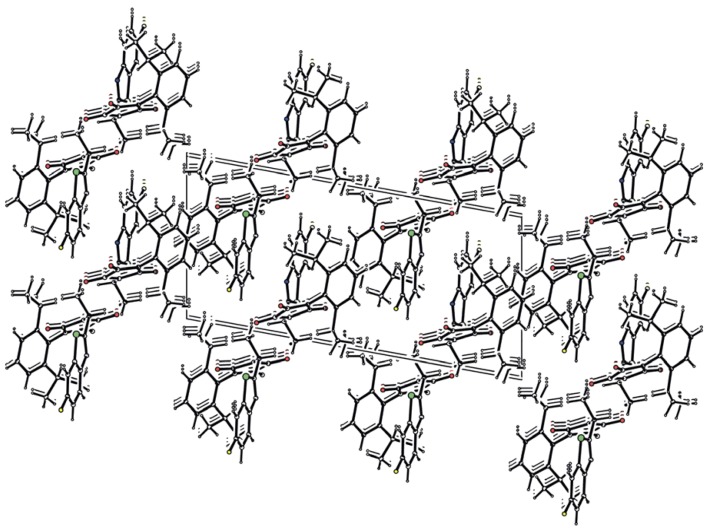
Supramolecular architecture of **3g**.

There is a quite extensive π-π stacking network causing the 3D structure formation with interactions between the heterocyclic rings of 3.372(3)Å and carbonyl groups of 3.121(3)Å, see Figure 2. Although one might expect very tight organization due to these interactions, remarkable cavities were found within the crystal lattice of compound **3g**, see Figure 1. No solvent can be accommodated in these cavities, probably because both hydrophilic and hydrophobic parts are present in the molecule of the mentioned compound. Another reason could be free rotability of the bulky diisopropylphenyl group. The π-π stacking interactions of aromatic rings and C=O groups in the molecules are shown in Figure 2. Molecules of compound **3g** also form interesting supramolecular architecture, see Figure 3.

### 2.3. Lipophilicity

Lipophilicity is a property that has a major effect on the absorption, distribution, metabolism, excretion and toxicity properties, as well as pharmacological activity, because drugs cross biological membranes through passive transport, which strongly depends on their lipophilicity. Lipophilicity has been studied and applied as an important drug property for decades [37]. Hydrophobicities (log *P*/Clog *P*) of the compounds were calculated using the commercially available program ChemOffice Ultra 11.0. The experimental partition coefficient log K_ow_ (*n*-octanol/water) was determined using the traditional shake-flask method. The *n*-octanol/water partition coefficient *P* (also referred to as K_ow_) is a measure of the propensity of a neutral compound to differentially dissolve in these immiscible phases. It is usually referred to as the logarithmic ratio, log *P*. The partition coefficient serves as a quantitative descriptor of lipophilicity and is one of the key determinants of pharmacokinetic properties. The partition coefficient log K_ow_ (*n*-octanol/water) indicates potential for crossing the blood-brain barrier for direct inhibition of brain cholinesterases [38]. Experimentally it is done by partitioning the molecule between water and the hydrophobic solvent *n*-octanol and determining the *P* value as the ratio of the concentration of the compound in *n*-octanol and in water. Log K_ow_ can be used as the lipophilicity index converted to *in silico* log *P* scale. The results are shown in Table 1.

**Table 1:** Comparison of calculated lipophilicities (log *P*/Clog *P*) with determined log K_ow_ values, Hammett’s σ parameters of prepared substituted imidazolidine-2,4,5-triones and their AChE and BChE inhibition in comparison with standards rivastigmine (RIV) and galanthamine (GLT). ChE inhibitions are expressed as mean ± SD (*n* = 3 experiments), and log K_ow_ data of the compounds are expressed as mean ± SD (*n* = 3 experiments).

Comp.	R	n	AChE	BChE	log K_ow_	log *P*/Clog *P*	σ [39]
			IC_50_ [μmol/L]				
**3a**	H	0	21.4 ± 0.19	14.5 ± 0.21	1.51 ± 0.03	3.91 / 2.769	0.00
**3b**	3-CF_3_	0	23.4 ± 0.28	17.4 ± 0.37	1.12 ± 0.15	4.83 / 3.652	0.43
**3c**	4-OCH_3_	0	22.4 ± 0.21	13.2 ± 0.22	0.69 ± 0.02	3.78 / 2.688	−0.27
**3d**	4-CH(CH_3_)_2_	0	16.6 ± 0.29	1.66 ± 0.14	1.64 ± 0.03	5.14 / 4.196	−0.15
**3e**	4-Cl	0	13.8 ± 0.13	25.7 ± 0.29	0.41 ± 0.02	4.46 / 3.482	0.23
**3f**	4-CN	0	19.1 ± 0.27	10.5 ± 0.18	0.41 ± 0.02	3.94 / 2.202	1.00
**3g**	2,6-CH(CH_3_)_2_	0	15.1 ± 0.29	30.2 ± 0.45	0.98 ± 0.08	6.38 / 5.623	0.06
**3h**	3-Cl-4-CH_3_	0	15.5 ± 0.2	17.0 ± 0.1	0.50 ± 0.02	4.95 / 3.981	0.20
**3i**	3,5-CH_3_	0	21.4 ± 0.19	6.76 ± 0.17	0.58 ± 0.03	4.88 / 3.767	−0.14
**3j**	3,5-Cl	0	17.0 ± 0.38	29.5 ± 0.39	0.77 ± 0.03	5.02 / 4.195	0.74
**3k**	H	1	18.6 ± 0.16	12.6 ± 0.11	1.06 ± 0.09	3.98 / 3.102	0.00
**RIV**	–	–	501 ± 3.08	19.95 ± 0.31	–	2.36 / 2.099	–
**GLT**	–	–	4.0 ± 0.13	7.96 ± 0.13	–	1.41 / 1.025	–

The results obtained with all the compounds show that the experimentally-determined lipophilicities (log K_ow_) of the discussed compounds are in poor accordance with the calculated values of compounds. This fact suggests significant intramolecular interactions within the whole series of the compounds. Compounds **3e** (4-Cl) and **4f** (4-CN) showed the lowest lipophilicity, while compounds **3a** (H) and **3d** [4-CH(CH_3_)_2_] demonstrated the highest. Benzyl derivative **3k** showed lower lipophilicity than phenyl derivative **3a**. Therefore it can be assumed that the determined log K_ow_ data specify lipophilicity within this series of the discussed compounds.

### 2.4. Inhibition of Cholinergic Enzymes

**Figure 4:**
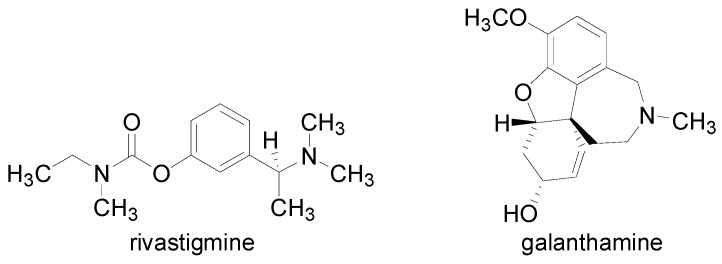
Structures of the two internal standards used, rivastigmine and galanthamine.

All the prepared carbamate-like compounds were tested for their inhibition of AChE and BChE. The activities of the compounds were compared with the internal standards rivastigmine (RIV, Exelon^®^) and galanthamine (GLT, Reminyl^®^), see Figure 4. These standards were chosen by reason of the different structures of both drugs. While rivastigmine is a classical acylating pseudo-reversible carbamate cholinesterase inhibitor that inhibits both acetylcholinesterase and butyrylcholinesterase, galanthamine is a non-acylating competitive reversible cholinesterase inhibitor and also an allosteric ligand at nicotinic acetylcholine receptors. The choice of these reference drugs with different mechanisms of action can provide relevant results. The results are summarized in Table 1 and expressed as 50% inhibitory concentration (IC_50_ [μmol/L]), or the concentration of inhibitor required for 50% inhibition of the mentioned enzymes.

The AChE inhibition of the prepared derivatives **3a**–**k** significantly exceeded the AChE inhibition of rivastigmine and these compounds are in average four-five times less efficient than the other standard galanthamine. Compound **3e** (4-Cl, IC_50_ = 13.8 μmol/L) showed the highest AChE-inhibiting activity. High inhibition was also expressed by compounds **3g** (2,6-CH(CH_3_)_2_, IC_50_ = 15.1 μmol/L), **3h** (3-Cl-4-CH_3_, IC_50_ = 15.5 μmol/L) and **3d** [4-CH(CH_3_)_2_, IC_50_ = 16.6 μmol/L]. The BChE inhibition of the prepared compounds (except compounds **3g**, **3j** and **3e**) also exceeded the BChE inhibition of rivastigmine. Only compound **3d** [4-CH(CH_3_)_2_, IC_50_ = 1.66 μmol/L] showed significantly higher BChE inhibition than galanthamine.

Based on these facts, it can be concluded that AChE is inhibited by *para*- and *meta*-substutited phenyl rings, while BChE is preferentially inhibited by a *para*-substutited phenyl ring. These observations describing steric/positional aspects of substitution on benzene are in agreement with recently published results by Chiou *et al.* indicating that that AChE prefers *para*- and *meta*-substitution to *ortho*-substitution, whereas BChE prefers *para*-substitution to *ortho*- and *meta*-substitution. These results imply that steric differences in the active sites of both enzymes can be found [40].

It is noteworthy that compounds with high inhibitory activity possess a branched substituent. For example, in case of the AChE inhibitors these are compounds **3d** [4-CH(CH_3_)_2_] and **3g** [2,6-CH(CH_3_)_2_] and in case of the BChE inhibitors it is compound **3d** [4-CH(CH_3_)_2_)].

**Figure 5:**
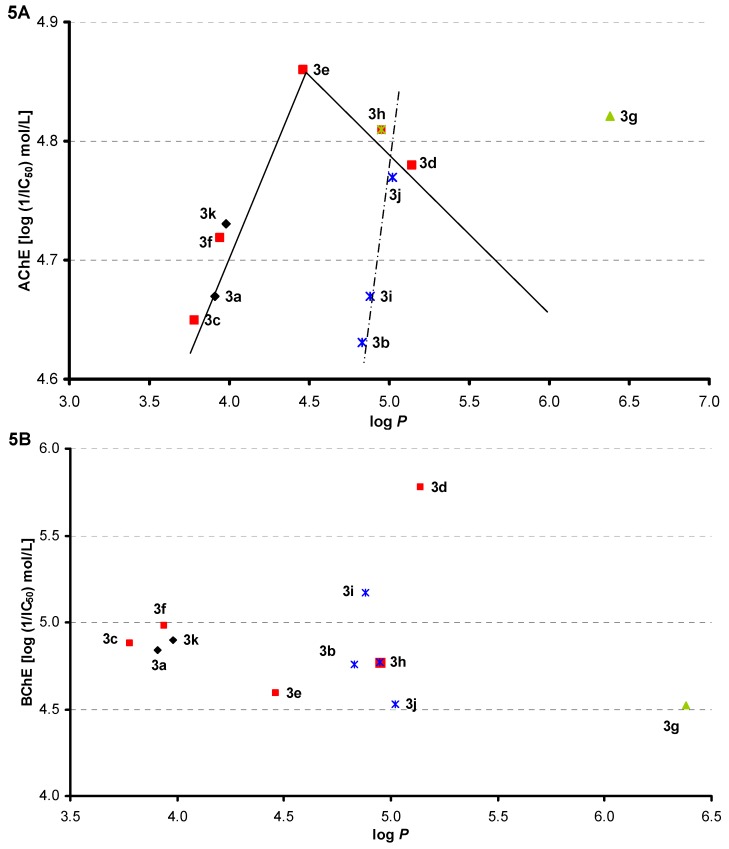
Dependence of AChE (**A**) and/or BChE (**B**) inhibition (log 1/IC_50_ [mol/L]) on compound lipophilicity expressed as log *P*.

The dependence of AChE inhibition (log L/IC_50_ [mol/L]) on log *P* is illustrated in Figure 5A. The set of 11 tested compounds can be divided into *para*-substituted and/or unsubstituted, where a bilinear dependence can be found, and *meta*-substituted, where activity sharply increases with a slight lipophilicity increase. The dependence of BChE inhibition (log L/IC_50_ [mol/L]) on log *P* is illustrated in Figure 5B, and from these relationships it is evident that lipophilicity is only a secondary parameter, although it seems that BChE inhibition activity decreases with lipophilicity increase within *meta*-substituted series.

**Figure 6:**
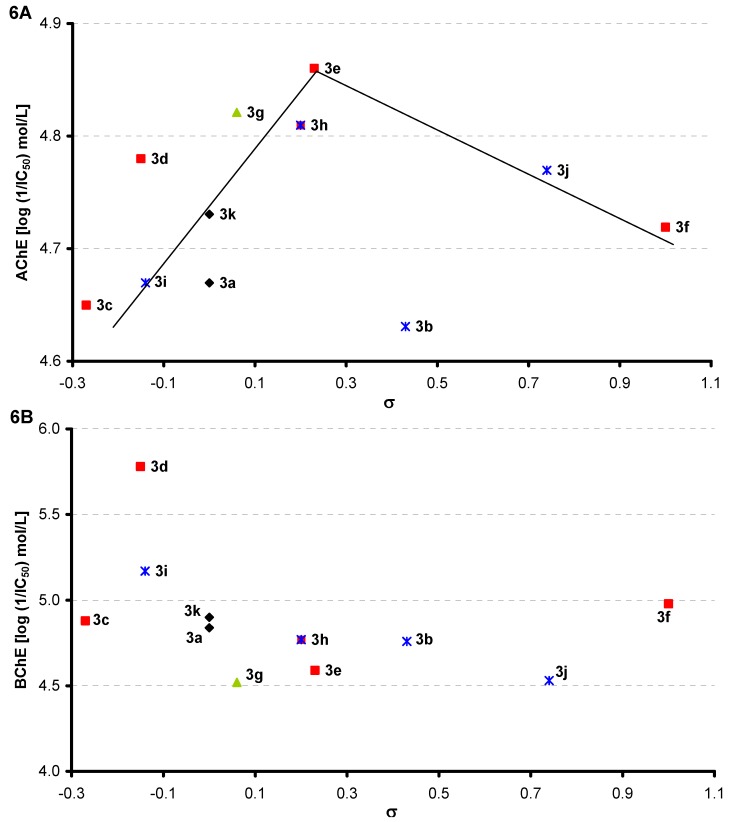
Dependence of AChE (**A**) and/or BChE (**B**) inhibition (log 1/IC_50_ [mol/L]) on substituent electron Hammett's σ parameters.

However, inhibition of cholinergic enzymes is also significantly dependent on the electronic properties of R phenyl substituents expressed as Hammett’s σ parameters. Figure 6A shows an evident general bilinear trend: with the increase of electron-withdrawing effect of individual substituents to the value σ = 0.23 (**3e**, 4-Cl), which is the optimum, AChE-inhibiting activity increases to the value IC_50_ = 13.8 μmol/L, and with the following increase of electron-withdrawing effect the activity decreases. Also other compounds **3g** [2,6-CH(CH_3_)_2_, σ = 0.06, IC_50_ = 15.1 μmol/L] and **3h** (3-Cl-4-CH_3_, σ = 0.20 IC_50_ = 15.5 μmol/L) with a close value of electron-donor substituent σ showed similar inhibition activity as **3e**.

In case of the BChE inhibitors the optimal value seems to be σ = −0.15 [**3d**, 4-CH(CH_3_)_2_, IC_50_ = 1.66 μmol/L] and with the following increase of electron-withdrawing effect the BChE-inhibiting activity decreases and is influenced minimally, see Figure 6B. The evident trend can be observed only within the *meta*-substituted series, where activity decreases with an increase of electron-withdrawing effect. Another compound with an electron-donor σ value similar to σ of compound **3d**, σ = −0.14 (**3i**, 3,5-CH_3_, IC_50_ = 6.76 μmol/L) is an inhibitor comparable with galanthamine. Also compounds with σ values close to σ = −0.15 (the Hammett’s parameter of the most effective compound **3d**) showed slightly lower BChE-inhibiting activity, see **3c** (σ = −0.27, IC_50_ = 13.2 μmol/L), **3a** (σ = 0.00, IC_50_ = 14.5 μmol/L), **3k** (σ = 0.00, IC_50_ = 12.6 μmol/L). On the other hand, the bulky disubstituted derivative **3g** [2,6-CH(CH_3_)_2_, σ = 0.06, IC_50_ = 30.2 μmol/L] possesses four times less inhibitory activity than the most active compound **3d**.

## 3. Experimental

### 3.1. General

All reagents and solvents were purchased from commercial sources (Sigma-Aldrich, Merck, Acros Organics, Lach-Ner CZ). Commercial grade reagents were used without further purification. The reactions were monitored and the purity of the products was checked by thin-layer chromatography plates coated with 0.2 mm silica gel 60 F_254_ (Merck, Darmstadt, Germany). TLC plates were visualized by UV irradiation (254 nm). All the melting points were determined on a Melting Point B-545 apparatus (Buchi, Germany) and are uncorrected. Infrared spectra (ZnSe ATR experiments) were recorded on a FT-IR spectrometer (Perkin Elmer, USA) in the range of 600–4000 cm^−1^. The NMR spectra were measured in DMSO-*d*_6_ solutions at ambient temperature on a Bruker Avance III 400 MHz spectrometer (Karlsruhe, Bruker, Germany, 400 MHz for ^1^H, 100 MHz for ^13^C and 376.5 MHz for ^19^F). Proton chemical shifts in DMSO-*d*_6_ are related to the middle of the solvent multiplet (δ = 2.50). ^13^C-NMR spectra were measured using APT pulse sequences. Carbon chemical shifts are referenced to the middle of the solvent multiplet (δ = 39.5 in DMSO-*d*_6_). ^19^F-NMR spectra were measured using waltz-16 proton decoupling and were standardised against fluorobenzene as the secondary external standard (δ = −113.1 against CFCl_3_ as the primary standard [41], td 64k zero filled to 128k). Chemical shifts for AB systems were calculated as weighed average of the line positions weighted by the peak intensities.

### 3.2. Synthesis

#### General Procedure for the Synthesis of Compounds **3a**–**k**

The appropriate 1-[(1*R*)-1-(6-fluoro-1,3-benzothiazol-2-yl)ethyl]-3-substituted phenyl urea **2a**–**k** (5 mmol) was added to a cooled solution (0–5 °C) of oxalyl chloride (7 mmol) in dichloromethane (20 mL) and mixture was stirred at temperature 0–5 °C for 1 h. The mixture was left to warm up to the ambient temperature and stirred for another 2 h. The reaction mixture was filtered and concentrated under reduced pressure. The addition of n-hexane caused precipitation of products **3a**–**k**. The precipitated solid was collected by filtration, dried in vacuum to give the title compounds in 80–85% yields as light yellow crystalline solids (unless stated otherwise).

*1-[(1R)-1-(6-Fluoro-1,3-benzothiazol-2-yl)ethyl]-3-phenylimidazolidine-2,4,5-trione* (**3a**). Yield: 85%; m.p. 156–157 °C; IR (cm^−1^): 3060, 2949, 1731, 1657, 1599, 1563, 1501,1453, 1404,1348, 1313, 1291, 1247, 1198, 1172, 1131, 1003, 947, 912, 836, 828, 757, 740, 686; ^1^H-NMR δ: 1.95 (d, 3H, 1-H, *J* = 7.2 Hz) CH_3_, 5.88 (q, 1H, 2-H, *J* = 7.2 Hz) CH, 7.41 (dt, 1H, 4-H, *J* = 2.7 Hz, *J* = 9.1 Hz, ^3^*J*(^19^F, ^1^H) = 9.1 Hz), 7.45–7.57 (m, 5H, 2´-H, 3´-H, 4´-H, 5´-H, 6´-H), 8.05 (dd, 1H, 6-H, *J* = 3.4 Hz, ^3^*J*(^19^F, ^1^H) = 9.0 Hz), 8.07 (d, 1H, 3-H, *J* = 8.9 Hz); ^13^C-NMR δ: 17.1, 49.1, 108.7 (d, ^2^*J*(^19^F, ^13^C) = 27.4 Hz), 115.0 (d, ^2^*J*(^19^F, ^13^C) = 24.9 Hz), 124.3 (d, ^3^*J*(^19^F, ^13^C) = 9.7 Hz), 126.7, 128.9, 129.2, 130.5, 136.5 (d, ^3^*J*(^19^F,^ 13^C) = 11.9 Hz), 148.8, 152.5, 156.0, 156.4, 159.8 (d, 1*J*(^19^F, ^13^C) = 243.1 Hz), 169.3 (d, ^4^*J*(^19^F, ^13^C) = 3.3 Hz); ^19^F-NMR δ: −115.67; Anal. Calcd. for C_18_H_12_FN_3_O_3_S (369.37): 58.53% C, 3.27% H, 11.38% N; found: 58.28% C, 3.38% H, 11.32%N.

*1-(3-Trifluoromethylphenyl)-3-[(1R)-1-(6-fluoro-1,3-benzothiazol-2-yl)ethyl]imidazolidine-2,4,5-trione* (**3b**). Yield: 80%; m.p. 145–146 °C; IR (cm^−1^): 3120, 2752, 1720, 1603, 1567, 1519, 1495, 1455, 1401, 1325, 1255, 1199, 1178, 1126, 1089, 1070, 1006, 953, 880, 913, 861, 829, 815, 791, 756, 695, 672, 658; ^1^H-NMR δ: 1.96 (d, 3H, 1-H, *J* = 7.2 Hz), 5.91 (q, 1H, 2-H, *J* = 7.2 Hz), 7.41 (dt, 1H, 4-H, *J* = 2.5 Hz, *J* = 9.0 Hz, ^3^*J*(^19^F, ^1^H) = 9.0 Hz), 7.82 (m, 4H, 2´-H, 4´-H, 5´-H,6´-H), 8.04 (dd, 1H, 6-H, *J* = 3.3 Hz, ^3^*J*(^19^F, ^1^H) = 8.8 Hz), 8.07 (d, 1H,3-H, *J* = 8.8 Hz); ^13^C-NMR δ: 17.1, 49.1, 108.6 (d, ^2^*J*(^19^F, ^13^C) = 27.1 Hz), 114.9 (d, ^2^*J*(^19^F, ^13^C) = 24.9 Hz), 123.2 (q, ^3^*J*(^19^F, ^13^C) = 4.0 Hz), 123.6 (q, ^1^*J*(^19^F, ^13^C) = 273.6 Hz), 124.2 (d, ^3^*J*(^19^F, ^13^C) = 9.3 Hz), 125.6 (q, ^3^*J*(^19^F, ^13^C) = 3.5 Hz), 129.8 (q, ^2^*J*(^19^F, ^13^C) = 32.4 Hz), 130.7, 130.8, 131.2, 136.5 (d, ^3^*J*(^19^F, ^13^C) = 11.7 Hz), 149.8 (d, ^5^*J*(^19^F, ^13^C) = 1.4 Hz), 152.1, 155.6, 156.1, 159.8 (d, ^1^*J*(^19^F, ^13^C) = 243.2 Hz), 169.0 (d, ^4^*J*(^19^F, ^13^C) = 3.2 Hz); ^19^F-NMR δ: −61.37 (s, 3F), −115.61 (s. 1F); Anal. Calcd. for C_19_H_11_F_4_N_3_O_3_S (437.37): 52.18% C, 2.54% H, 9.61% N; found: 52.29% C, 2.48% H, 9.66% N.

*1-(4-Methoxyphenyl)-3-[(1R)-1-(6-fluoro-1,3-benzothiazol-2-yl)ethyl]imidazolidine-2,4,5-trione* (**3c**). Yield: 83%, m.p. 79–80 °C; IR (cm^−1^): 3075, 2934, 1735, 1604, 1566, 1514, 1454, 1398, 1331, 1303, 1257, 1197, 1172, 1131, 1024, 938, 911, 824, 753, 701; ^1^H-NMR δ: 1.94 (d, 3H, 1-H, *J* = 7.2 Hz), 3.80 (s, 3H) OCH_3_, 5.87 (q, 1H, 2-H, *J* = 7.2 Hz), 7.08 (d, 2H, 2´-H, 6´-H, *J* = 8.8 Hz), 7.34 (d, 2H, 3´-H, 5´-H, *J* = 8.8 Hz), 7.41 (dt, 1H, 4-H, *J* = 2.8 Hz, *J* = 9.2 Hz, ^3^*J*(^19^F, ^1^H) = 9.2 Hz), 8.05 (d, 1H, 3-H, *J* = 8.9 Hz) 8.07 (dd, 1H, 6-H, *J* = 3.3 Hz, ^3^*J*(^19^F, ^1^H) = 8.7 Hz); ^13^C-NMR δ: 17.1, 49.0, 55.4, 108.7 (d, ^2^*J*(^19^F, ^13^C) = 27.2 Hz), 114.4, 114.9 (d, ^2^*J*(^19^F, ^13^C) = 24.9 Hz), 122.9, 124.2 (d, ^3^*J*(^19^F, ^13^C) = 9.7 Hz), 128.2, 136.5 (d, ^3^*J*(^19^F, ^13^C) = 11.9 Hz), 148.8, 152.8, 156.2, 156.5, 159.3, 159.8 (d, ^1^*J*(^19^F, ^13^C) = 243.2 Hz), 169.3 (d, ^4^*J*(^19^F, ^13^C) = 3.2 Hz); ^19^F-NMR δ: −115.67; Anal. Calcd. for C_19_H_14_FN_3_O_4_S (399.40): 57.14% C, 3.53% H, 10.52% N; found: 57.25% C, 3.49% H, 10.60% N.

*1-(4-Isopropylphenyl)-3-[(1R)-1-(6-fluoro-1,3-benzothiazol-2-yl)ethyl]imidazolidine-2,4,5-trione* (**3d**). Yield: 80%; m.p. 70–71 °C; IR (cm^−1^): 3061, 2955, 1734, 1602, 1567, 1515, 1454, 1397, 1342, 1248, 1199, 1134, 1019, 937, 912, 840, 816, 754, 654; ^1^H-NMR δ: 1.22 (d, 6H, CH_3_-isopropyl, *J* = 6.8 Hz), 1.96 (d, 3H, 1-H, *J* = 7.2 Hz), 2.94 (sept., 1H, CH-isopropyl, *J* = 7.2 Hz), 5.87 (q, 1H, 2-H, *J* = 7.2 Hz), 7.34 (d, 2H, 2´-H, 6´-H, *J* = 8.3 Hz), 7.40 (d, 2H, 3´-H, 5´-H, *J* = 8.3 Hz), 7.41 (dt, 1H, 4-H, *J* = 2.8 Hz, *J* = 9.2 Hz, ^3^*J*(^19^F, ^1^H) = 9.2 Hz), 8.05 (d, 1H, 3-H, *J* = 8.9 Hz), 8.07 (dd, 1H, 6-H, *J* = 2.6 Hz, ^3^*J*(^19^F, ^1^H) = 8.7 Hz); ^13^C-NMR δ: 17.0, 23.7, 33.2, 49.0, 108.6 (d, ^2^*J*(^19^F, ^13^C) = 27.3 Hz), 114.9 (d, ^2^*J*(^19^F, ^13^C) = 24.9 Hz), 124.2 (d, ^3^*J*(^19^F, ^13^C) = 9.4 Hz), 126.6, 127.0, 128.0, 136.5 (d, ^3^*J*(^19^F, ^13^C) = 11.8 Hz), 148.8, 149.3,152.6, 156.0, 156.4, 159.8 (d, ^1^*J*(^19^F, ^13^C) = 243.3 Hz), 169.3 (d, ^4^*J*(^19^F, ^13^C) = 3.4 Hz); ^19^F-NMR δ: −115.73; Anal. Calcd. for C_21_H_18_FN_3_O_3_S (411,45): 61.30% C, 4.41% H, 10.21% N; found: 61.55% C, 4.33% H, 10.33% N.

*1-(4-Chlorophenyl)-3-[(1R)-1-(6-fluoro-1,3-benzothiazol-2-yl)ethyl]imidazolidine-2,4,5-trione* (**3e**). Yield: 85%; m.p. 149–150 °C; IR (cm^−1^): 3060, 2938, 1737, 1600, 1564, 1516, 1494, 1455, 1401, 1350, 1311, 1290, 1248, 1198, 1176, 1128, 1091, 1017, 1002, 950, 911, 838, 849, 821, 750, 664, 714, 699, 655; ^1^H-NMR δ: 1.93 (d, 3H, 1-H, *J* = 7.2 Hz), 5.86 (q, 1H, 2-H, *J* = 7.2 Hz), 7.41 (dt, 1H, 4-H, *J* = 2.8 Hz, *J* = 9.2 Hz, ^3^*J*(^19^F, ^1^H) = 9.2 Hz), 7.46 (d, 2H, 2´-H, 6´-H, *J* = 9.2 Hz), 7.62 (d, 2H, 3´-H, 5´-H, *J* = 9.0 Hz), 8.04 (dd, 1H, 6-H, *J* = 2.8 Hz, ^3^*J*(^19^F, ^1^H) = 8.6 Hz), 8.06 (d, 1H, 3-H, *J* = 8.8 Hz); ^13^C-NMR δ: 17.0, 48.9, 108.6 (d, ^2^*J*(^19^F, ^13^C) = 27.2 Hz), 114.9 (d, ^2^*J*(^19^F, ^13^C) = 24.8 Hz), 124.2 (d, ^3^*J*(^19^F, ^13^C) = 9.8 Hz), 128.6, 129.4, 129.8, 133.5 136.6 (d, ^3^*J*(^19^F, ^13^C) = 11.9 Hz), 148.9, 152.4, 155.9, 156.4, 159.5 (d, ^1^*J*(^19^F, ^13^C) = 243.0 Hz), 169.3 (d, ^4^*J*(^19^F, ^13^C) = 3.3 Hz); ^19^F-NMR δ: −115.55; Anal. Calcd. for C_18_H_11_ClFN_3_O_3_S (403.81): 53.54% C, 2.75% H, 10.41% N; found: 53.22% C, 3.80% H, 10.50% N.

*1-(4-Cyanophenyl)-3-[(1R)-1-(6-fluoro-1,3-benzothiazol-2-yl)ethyl]imidazolidine-2,4,5-trione* (**3f**). A white crystalline compound; Yield: 80%; m.p. 172–173 °C; IR (cm^−1^): 3071, 1739, 1653, 1602, 1564, 1508, 1455, 1397, 1355, 1314, 1249, 1207, 1136, 1064, 996, 940, 913, 842, 817, 750, 691, 667; ^1^H-NMR δ: 1.94 (d, 3H, 1-H, *J* = 7.2 Hz), 5.90 (q, 1H, 2-H, *J* = 7.2 Hz), 7.41 (dt, 1H, 4-H, *J* = 2.8 Hz, *J* = 9.2 Hz, ^3^*J*(^19^F, ^1^H) = 9.2 Hz), 7.65 (d, 2H, 2´-H, 6´-H, *J* = 8.4 Hz), 8.00 (d, 2H, 3´-H, 5´-H, *J* = 8.4 Hz), 8.04 (dd, 1H, 6-H, *J* = 2.9 Hz, ^3^*J*(^19^F, ^1^H) = 8.8 Hz), 8.06 (d, 1H, 3-H, *J* = 8.9 Hz); ^13^C-NMR δ: 17.0, 49.2, 108.7 (d, ^2^*J*(^19^F, ^13^C) = 27.3 Hz), 111.2, 115.0 (d, ^2^*J*(^19^F, ^13^C) = 24.9 Hz), 118.2, 124.3 (d, ^3^*J*(^19^F, ^13^C) = 9.6 Hz), 126.9, 133.4, 134.5, 136.5 (d, ^3^*J*(^19^F, ^13^C) = 11.8 Hz), 148.8, 151.9, 155.3, 156.0, 159.8 (d, ^1^*J*(^19^F, ^13^C) = 243.0 Hz), 169.0 (d, ^4^*J*(^19^F, ^13^C) = 3.2 Hz); ^19^F-NMR δ: −115.61; Anal. Calcd. for C_19_H_11_FN_4_O_3_S (394,38): 57.86% C, 2.81% H, 14.21% N; found: 57.68% C, 2.90% H, 14.08% N.

*1-(2,6-Diisopropylphenyl)-3-[(1R)-1-(6-fluoro-1,3-benzothiazol-2-yl)ethyl]imidazolidine-2,4,5-trione* (**3g**). Yield: 81%; m.p. 165–166 °C; IR (cm^−1^): 3060, 2951, 2809, 1739, 1602, 1567, 1458, 1395, 1364, 1295, 1251, 1216, 1194, 1139, 1034, 1005, 960, 911, 853, 846, 811, 793, 755, 741, 710; ^1^H-NMR δ: 1.04 (b, 3H, CH_3_-isopropyl, *J* = 6.7 Hz), 1.09 (d, 3H, CH_3_-isopropyl, *J* = 6.7 Hz), 1.14 (d, 3H, CH_3_‑isopropyl, *J* = 6.7 Hz), 1.16 (d, 3H, CH_3_-isopropyl, *J* = 6.7 Hz), 2.01 (d, 3H, 1-H, *J* = 7.2 Hz), 3.00 (m, 2H, CH-isopropyl, *J* = 6.7 Hz), 5.89 (q, 1H, 2-H, *J* = 7.2 Hz), 7.33–7.36 (m, 3´-H, 5´-H), 7.42 (dt, 1H, 4-H, *J* = 2.7 Hz, *J* = 9.2 Hz, ^3^*J*(^19^F, ^1^H) = 9.2 Hz), 7.97 (dd, 1H, 6-H, *J* = 4.8 Hz, ^3^*J*(^19^F, ^1^H) = 9.0 Hz), 8.08 (dd, 4-H, *J* = 9.2 Hz, ^4^*J*(^19^F, ^1^H) = 2.8 Hz); ^13^C-NMR δ: 17.0, 23.7, 23.8, 23.9, 24.0, 28.1, 49.3, 108.8 (d, ^2^*J*(^19^F, ^13^C) = 27.6 Hz), 115.2 (d, ^2^*J*(^19^F, ^13^C) = 25.3 Hz), 124.0 (d, ^3^*J*(^19^F, ^13^C) = 9.2 Hz), 124.3, 125.5, 130.8, 136.3 (d, ^3^*J*(^19^F, ^13^C) = 11.8 Hz), 147.1 (d, ^5^*J*(^19^F, ^13^C) = 1.5 Hz), 148.8, 153.0, 156.5, 156.8, 159.8 (d, ^1^*J*(^19^F, ^13^C) = 242.9 Hz), 169.8 (d, ^4^*J*(^19^F, ^13^C) = 2.6 Hz); ^19^F-NMR δ: −115.63; Anal. Calcd. for C_24_H_24_FN_3_O_3_S (453.53): 63.56% C, 5.33% H, 9.27% N; found: 63.33 C, 5.40% H, 9.20% N.

*1-(3-Chloro-4-methylphenyl)-3-[(1R)-1-(6-fluoro-1,3-benzothiazol-2-yl)ethyl]imidazolidine-2,4,5-trione* (**3h**). Yield: 85%; m.p. 161–162 °C; IR (cm^−1^): 3029, 1724, 1600, 1565, 1498, 1457, 1382, 1412, 1396, 1339, 1246, 1217, 1195, 1142, 1096, 1057, 1008, 960, 890, 847, 816, 757, 696, 681, 661; ^1^H-NMR δ: 1.95 (d, 3H, 1-H, *J* = 7.5 Hz), 2.38 (s, 3H, CH_3_), 5.89 (q, 1H, 2-H, *J* = 7.2 Hz), 7.34 (dd, 1H, 6´-H, *J* = 2.0 Hz, *J* = 8.0 Hz), 7.40 (dt, 1H, 4-H, *J* = 2.4 Hz, *J* = 9.2 Hz, ^3^*J*(^19^F, ^1^H) = 9.2 Hz), 7.51 (d, 1H, 2´-H, *J* = 2.4 Hz), 7.53 (d, 1H, 5´-H, *J* = 8.0 Hz), 8.04 (d, 1H, 3-H, *J* = 8.8 Hz), 8.06 (dd, 1H, 6-H, *J* = 3.2 Hz, ^3^*J*(^19^F, ^1^H) = 8.8 Hz); ^13^C-NMR δ: 17.0, 19.3, 49.1, 108.6 (d, ^2^*J*(^19^F, ^13^C) = 27.2 Hz), 114.9 (d, ^2^*J*(^19^F, ^13^C) = 24.8 Hz), 124.2 (d, ^3^*J*(^19^F, ^13^C) = 9.7 Hz), 125.4, 126.7, 129.2, 131.6, 133.2, 135.5 (d, ^3^*J*(^19^F, ^13^C) = 11.8 Hz), 136.6, 148.8 (d, ^5^*J*(^19^F, ^13^C) = 2.0 Hz), 152.2, 155.7, 156.2, 159.8 (d, ^1^*J*(^19^F, ^13^C) = 243.1 Hz), 169.1 (d, ^4^*J*(^19^F, ^13^C) = 3.0 Hz); ^19^F-NMR δ: −115.59; Anal. Calcd. for C_19_H_13_ClFN_3_O_3_S (417.84): 54.61% C, 3.14% H, 10.06% N; found: 54.75% C, 3.08% H, 10.11% N.

*1-(3,5-Dimethylphenyl)-3-[(1R)-1-(6-fluoro-1,3-benzothiazol-2-yl)ethyl]urea* (**3i**). Yield: 83%; m.p. 122–123 °C; IR (cm^−1^): 3004, 1731, 1604, 1566, 1520, 1455, 1400, 1379, 1345, 1279, 1256, 1206, 1161, 1098, 1010, 963, 919, 905, 858, 844, 825, 811, 756, 683, 654; ^1^H-NMR δ: 1.94 (d, 3H, 1-H, *J* = 7.2 Hz), 2.32 (s, 6H, CH_3_), 5.86 (q, 1H, 2-H, *J* = 7.2 Hz), 7.03 (s, 2H, 2´-H, 6´-H), 7.11 (s, 1H, 4´-H), 7.41 (dt, 1H, 4-H, *J* = 2.8 Hz, *J* = 9.2 Hz, ^3^*J*(^19^F, ^1^H) = 9.2 Hz), 8.04 (dd, 1H, 6-H, *J* = 3.2 Hz, ^3^*J*(^19^F, ^1^H) = 8.8 Hz), 8.07 (d, 1H, 3-H, *J* = 8.9 Hz); ^13^C-NMR δ: 17.0, 20.7, 49.0, 108.7 (d, ^2^*J*(^19^F, ^13^C) = 27.3 Hz), 114.9 (d, ^2^*J*(^19^F, ^13^C)) = 24.9 Hz), 124.2, 124.3 (d, ^3^*J*(^19^F, ^13^C) = 9.5 Hz), 130.2, 130.3, 136.5 (d, ^3^*J*(^19^F, ^13^C) = 11.7 Hz), 138.5, 148.8, 152.5, 156.0, 1156.4, 159.8 (d, ^1^*J*(^19^F, ^13^C) = 243.0 Hz), 169.4 (d, ^4^*J*(^19^F, ^13^C) = 3.0 Hz); ^19^F-NMR δ: −115.67; Anal. Calcd. for C_20_H_16_FN_3_O_3_S (397.42): 60.44% C, 4.06% H, 10.57% N; found: 60.56% C, 3.96% H, 10.59% N.

*1-(3,5-Dichlorophenyl)-3-[(1R)-1-(6-fluoro-1,3-benzothiazol-2-yl)ethyl]imidazolidine-2,4,5-trione* (**3j**). Yield: 83%; m.p. 190–191 °C; IR (cm^−1^): 3130, 2950, 1727, 1566, 1573, 1590, 1519, 1452, 1432, 1406, 1381, 1342, 12454, 1205, 1196, 1173, 1129, 1105, 1010, 966, 916, 857, 828, 817, 757, 726, 674, 664; ^1^H-NMR δ: 1.94 (d, 3H, 1-H, *J* = 6.8 Hz), 5.89 (q, 1H, 2-H, *J* = 6.8 Hz), 7.41 (dt, 1H, 4-H, *J* = 2.4 Hz, *J* = 9.2 Hz, ^3^*J*(^19^F, ^1^H) = 9.2 Hz), 7.52 (d, 2H, 2´-H, 6´-H, *J* = 2.0 Hz), 7.80 (t, 1H, 4´-H, *J* = 2.0 Hz), 8.04 (d, 1H, 3-H, *J* = 8.4 Hz), 8.06 (dd, 1H, 6-H, *J* = 3.2 Hz, ^3^*J*(^19^F, ^1^H) = 8.8 Hz); ^13^C-NMR δ: 17.0, 49.2, 108.7 (d, *J*(^19^F, ^13^C) = 27.2 Hz), 115.0 (d, ^2^*J*(^19^F, ^13^C) = 24.8 Hz), 124.3 (d, ^3^*J*(^19^F, ^13^C) = 9.8 Hz), 125.3, 128.7, 132.5, 134.4, 136.5 (d, ^3^*J*(^19^F, ^13^C) = 11.6 Hz), 148.8, 151.8, 155.2, 155.9, 159.5 (d, ^1^*J*(^19^F, ^13^C) = 243.1 Hz), 168.9 (d, ^4^*J*(^19^F, ^13^C) = 3.3 Hz); ^19^F-NMR δ: −115.55; Anal. Calcd. for C_18_H_10_Cl_2_FN_3_O_3_S (438.26): 49.33% C, 2.30% H, 9.59% N; found: 49.49% C, 2.26% H, 9.66% N.

*1-Benzyl-3-[(1R)-1-(6-fluoro-1,3-benzothiazol-2-yl)ethyl]imidazolidine-2,4,5-trione* (**3k**). Yield: 84%; m.p. 193–195 °C; IR (cm^−1^): 3035, 2950, 1721, 1603, 1566, 1493, 1456, 1440, 1413, 1397, 1349, 1305, 1251, 1201, 1191, 1133, 1073, 1025, 858, 818, 755, 700, 680, 665; ^1^H-NMR δ: 1.90 (d, 3H, 1-H, *J* = 7.2 Hz), 4.73 (s, 2H, CH_2_-benzyl), 5.80 (q, 1H, 2-H, *J* = 7.2 Hz), 5.6 (t, 1H, 4´-H, *J* = 6.0 Hz), 7.31‑7.38 (m, 5H, 2´-H, 3´-H, 4´-H, 5´-H, 6´-H), 7.40 (dt, 1H, 4-H, *J* = 2.8 Hz, *J* = 8.8 Hz, ^3^*J*(^19^F, ^1^H) = 8.8 Hz), 8.04 (dd, 1H, 6-H, *J* = 3.3 Hz, ^3^*J*(^19^F, ^1^H) = 8.9 Hz), 8.07 (d, 1H, 3-H, *J* = 8.8 Hz); ^13^C-NMR δ: 17.1, 42.0, 48.9, 108.7 (d, ^2^*J*(^19^F, ^13^C) = 27.2 Hz), 114.9 (d, ^2^*J*(^19^F, ^13^C) = 24.9 Hz), 124.2 (d, ^3^*J*(^19^F, ^13^C) = 9.7 Hz), 126.7,127.7, 128.6, 135.3 136.4 (d, ^3^*J*(^19^F, ^13^C) = 11.6 Hz), 148.8, 153.4, 156.7, 156.9, 159.8 (d, ^1^*J*(^19^F, ^13^C) = 243.0 Hz), 169.5 (d, ^4^*J*(^19^F, ^13^C) = 3.9 Hz); ^19^F-NMR δ: −115.71; Anal. Calcd. for C_19_H_14_FN_3_O_3_S (383.40): 59.52% C, 3.68% H, 10.96% N; found: 59.65% C, 3.60% H, 11.02% N.

### 3.3. Determination of Crystallography

The X-Ray data for colourless crystals of compound **3g** were obtained at 150 K using Oxford Cryostream low-temperature device on a Nonius KappaCCD diffractometer with MoK_α_ radiation (λ = 0.71073 Å), a graphite monochromator and the 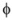 and χ scan mode. Data reductions were performed with DENZO-SMN [42]. The absorption was corrected by integration methods [43]. Structures were solved by direct methods (Sir92) [44] and refined by full matrix least-square based on *F^2^* (SHELXL97) [45]. Hydrogen atoms were mostly localized on a difference Fourier map, however to ensure uniformity of the treatment of the crystal, all hydrogen atoms were recalculated into idealized positions (riding model) and assigned temperature factors H_iso_(H) = 1.2 U_eq_(pivot atom) or of 1.5 U_eq_ for the methyl moiety with C–H = 0.96, 0.98 and 0.93 Å for methyl, methine and hydrogen atoms in the aromatic rings, respectively.

Selected crystallographic data for compound **3g**: C_24_H_24_FN_3_O_3_S, M = 453.52, triclinic, *P-1*, *a* = 10.1760(7), *b* = 10.7150(6), *c* = 21.5609(18)Å, α = 99.229(6), β = 100.256(8), γ = 91.188(6), Z = 4, V = 2280.5(3) Å^3^, D_c_ = 1.321 g/cm^3^, μ = 0.181 mm^−1^, T_min_ = 0.959, T_max_ = 0.986; 41644 reflections measured (θ_max_ = 27.5°), 10323 independent (R_int_ = 0.0630), 6104 with *I* >* 2σ(I)*, 577 parameters, *S* = 1.079, *R_1_*(obs. data) = 0.0915, *wR_2_*(all data) = 0.1922; max., min. residual electron density = 1.295, −0.538eǺ^−3^. *R*_int_ = Σ |*F*_o_^2^‑*F*_o,mean_^2^| / Σ *F*_o_^2^, GOF = [Σ (*w*(*F*_o_^2^ − *F*_c_^2^)^2^) / (*N*_diffrs_ − *N*_params_)]^½^ for all data, *R*(*F*) = Σ| |*F*_o_| − |*F*_c_| | / Σ |*F*_o_| for observed data, *wR*(*F*^2^) = [Σ (*w*(*F*_o_^2^ − *F*_c_^2^)^2^) / (Σ *w*(*F*_o_^2^)^2^)]^½^ for all data.

Crystallographic data for structural analysis have been deposited with the Cambridge Crystallographic Data Centre (deposition number CCDC 837618). Copies of this information may be obtained free of charge from the Director, CCDC, 12 Union Road, Cambridge CB2 1EY, UK (fax: +44-1223-336033; e-mail: deposit@ccdc.cam.ac.uk or http://www.ccdc.cam.ac.uk).

### 3.4. Determination of Partition Coefficient K_ow_

Before the partition coefficient is determined, the two solvents are mutually saturated at the temperature of the experiment. To do this, it is practical to shake two large stock bottles, one containing *n*-octanol and a sufficient quantity of water, and the other containing water and a sufficient quantity of *n*-octanol, for 24 hours on a mechanical shaker and then to let them stand long enough to allow the phases to separate [46].

The procedure of determination was the following: *n*-Octanol (2 mL) was placed in a test tube. Then an octanol solution of the chosen inhibitor (15 μL, 0.01 M) was added. Mixture was intensively shaken for 15 min. This mixture (1 mL) was placed into the cell, and its absorbance at the absorption maximum wavelength was measured. The reference solution was *n*-octanol. The value of absorbance corresponding to 100% of the chosen inhibitor in *n*-octanol was obtained. An *n*-octanol solution of the chosen inhibitor (0.01 M, 15 μL) was added into the mixture of *n-*octanol and water (1:1, total volume 4 mL). The mixture was intensively shaken for 15 min and then centrifuged (3,000 rpm, 10 min). One mL of the *n*-octanol layer was put into the cell, and its absorbance at the wavelength of absorption maximum was measured. The comparative solution was *n*-octanol again. The percentage content of chosen inhibitor in the octanol layer (%) was obtained. The *n*-octanol/water partition coefficient is defined as *P*_ow_ = c_1_/c_2_, where c_1_ and c_2_ are molar concentrations of tested compounds in *n*-octanol and water. For each compound, at least three determinations were performed. The log K_ow_ values of the individual compounds are shown in Table 1.

### 3.5. Lipophilicity Calculations

Log *P*, *i.e.*, the logarithm of the partition coefficient for *n*-octanol/water, was calculated using the program CS ChemOffice Ultra ver. 11.0 (CambridgeSoft, Cambridge, MA, USA). Clog *P* values (the logarithm of *n*-octanol/water partition coefficient based on established chemical interactions) were generated by means of the same software. The results are shown in Table 1.

### 3.6. In Vitro Evaluation of AChE- and BChE-Inhibiting Activity

The ability of all tested compounds to inhibit acetylcholinesterase from electric eel (*Electrophorus electricus* L.) and butyrylcholinesterase from equine serum (both purchased from Sigma) was tested. The effectiveness of the inhibitor could be described by the 50% inhibitory concentration IC_50_. The IC_50_, or the half maximal inhibitory concentration, represents the concentration of an inhibitor that is required for 50% inhibition of the enzyme (sometimes it is referred to as the negative logarithm of the molar concentration inhibiting the enzyme activity by 50%, pI_50_ = log 1/IC_50_). The IC_50_ values were determined by the spectrophotometric Ellman’s method.

The Ellman’s method is a simple, rapid and direct method to determine the SH and –S–S– group content in proteins [47]. This method is widely used for measuring of cholinesterase activity and effectivity of cholinesterase inhibitors. Cholinesterase activity is measured indirectly by quantifying the concentration of 5-thio-2-nitrobenzoic acid (TNB) ion formed in the reaction between the thiol reagent 5,5′-dithiobis-2-nitrobenzoic acid (DTNB) and thiocholine, a product of substrate (*i.e.*, acetylthiocholine, ATCh) hydrolysis by the cholinesterase [48]. All tested compounds were dissolved in dioxane (concentration 0.01 M) and then diluted in demineralized water (concentration 0.001 M and 0.0001 M). The procedure of determination of IC_50_ is in detail described in [49]. For determination of IC_50_ values the inhibition was determined at 12 different compound concentrations with three replicates. The obtained results are summarized in Table 1.

## 4. Conclusions

A series of eleven original 2-substituted benzothiazole derivates incorporating 1,3-disubstituted imidazolidine-2,4,5-triones were synthesized and characterized. Their octanol/water partition coefficients were determined experimentally as a basic property of compounds crossing biological membranes through passive transport. Their ability to inhibit cholinergic enzymes (AChE and BChE) was tested using Ellman’s method. The determined IC_50_ for each prepared compound was also compared with the inhibitory activity of the internal standards rivastigmine and galanthamine. All the discussed carbamate-like compounds expressed significantly higher AChE inhibitory activity than the standard rivastigmin and slightly lower inhibitory activity than the standard galanthamine. Almost half of the prepared compounds are stronger BChE inhibitors than both the internal standards used, galanthamine and rivastigmine. 1-(4-chlorophenyl)-3-[(1*R*)-1-(6-fluoro-1,3-benzothiazol-2-yl)ethyl]-imidazolidine-2,4,5-trione (**3e**, IC_50_ = 13.8 μmol/L) showed the highest AChE-inhibiting activity within the series and 1-(4-isopropylphenyl)-3-[(1*R*)-1-(6-fluoro-1,3-benzothiazol-2-yl)ethyl]-imidazolidine-2,4,5-trione (**3d**, IC_50_ = 1.66 μmol/L) expressed the highest BChE-inhibiting activity, which is almost five times higher the that activity of galanthamine. Substitution of phenyl in the position *para* is advantageous for inhibitory activity, especially in case of BChE inhibitors. It was also found that inhibition of both cholinergic enzymes is more connected with electron properties of individual substituents on phenyl than with lipophilicity of the discussed compounds. A branched substituent on the phenyl ring positively influenced cholinergic inhibitory activity.

## Supplementary Materials

Supplementary File 1
